# Current-Assisted SPAD with Improved p-n Junction and Enhanced NIR Performance

**DOI:** 10.3390/s20247105

**Published:** 2020-12-11

**Authors:** Gobinath Jegannathan, Thomas Van den Dries, Maarten Kuijk

**Affiliations:** Department of Electronics and Informatics (ETRO), Vrije Universiteit Brussel, 1050 Brussels, Belgium; Thomas.Van.Den.Dries@vub.be (T.V.d.D.); mkuijk@vub.be (M.K.)

**Keywords:** current-assistance, SPAD, CMOS, Geiger mode, single photon detector, avalanche breakdown

## Abstract

Single-photon avalanche diodes (SPADs) fabricated in conventional CMOS processes typically have limited near infra-red (NIR) sensitivity. This is the consequence of isolating the SPADs in a lowly-doped deep N-type well. In this work, we present a second improved version of the “current-assisted” single-photon avalanche diode, fabricated in a conventional 350 nm CMOS process, having good NIR sensitivity owing to 14 μm thick epilayer for photon absorption. The presented device has a photon absorption area of 30 × 30 µm^2^, with a much smaller central active area for avalanche multiplication. The photo-electrons generated in the absorption area are guided swiftly towards the central area with a drift field created by the “current-assistance” principle. The central active avalanche area has a cylindrical p-n junction as opposed to the square geometry from the previous iteration. The presented device shows improved performance in all aspects, most notably in photon detection probability. The p-n junction capacitance is estimated to be ~1 fF and on-chip passive quenching with source followers is employed to conserve the small capacitance for bringing monitoring signals off-chip. Device physics simulations are presented along with measured dark count rate (DCR), timing jitter, after-pulsing probability (APP) and photon detection probability (PDP). The presented device has a peak PDP of 22.2% at a wavelength of 600 nm and a timing jitter of 220 ps at a wavelength of 750 nm.

## 1. Introduction

Single-photon avalanche diodes (SPADs), due to their single photon sensitivity, have been employed in many photon-starved applications such as fluorescence lifetime imaging (FLI), time-of-flight (TOF) and conventional imaging [1,2,3,4,5,6,7]. The possibility of fabricating SPADs in a conventional CMOS process facilitates mass production with lower associated costs. Nevertheless, special features and variations to the conventional process are often needed to improve performance of the SPADs. CMOS compatibility also facilitates having integrated circuits on the same chip, which can be used for readout and to process the data.

A SPAD is a reverse-biased p-n junction diode biased at a larger voltage than its reverse breakdown voltage. At this bias, the diode is in a meta-stable state called “Geiger regime” where a single photo-carrier can lead to multiple secondary carriers and secondary carriers each generate multiple carriers and so on, resulting in a large self-sustainable avalanche current. As such, a single photo-carrier can generate a large enough signal to be detected. The self-sustaining avalanche current has to be stopped to avoid permanent damage to the junction. This is done by “quenching”. Quenching and subsequent recharging of the junction is also needed for rearming the SPAD to detect the next photon. Various quenching techniques exist, and each have their merits and demerits [8,9].

One of the features of the front-side illuminated (FSI) SPAD is that the active area where the avalanche breakdown happens is almost the same as the photon-detection area. Some photo-carriers are generated slightly deeper than the active area in the depleted region and are guided to the active area with the depletion drift field. But the photo-carriers which are generated outside the depletion region either slowly diffuse towards the active region, adding an exponential tail in the timing response or simply recombine, resulting to a drop in photon detection probability. Therefore, simply increasing the absorption region thickness does not equate to increased sensitivity if there is no drift field and the main transport mechanism of the photo-generated carriers is diffusion. This can also lead to crosstalk between two adjacent SPADs because diffusion is not directional.

Previously, we have reported a proof-of-concept current-assisted SPAD (referred to as CA-SPAD-1 [10]) which had a large absorption volume with drift field generated through current-assistance. In short, when there is a majority current flow (holes in p-type) due to a potential difference, the minority carriers (electrons in our case) also experience a drift field in the direction opposite to that of the majority hole current. This “current-assisted” principle has been used successfully in applications such as time-of-flight (TOF) [11], fluorescence lifetime imaging (FLI) [12] and high-speed optical receivers (CAD) [13]. In the past, we have also demonstrated the current-assisted avalanche photodiodes [14] and current-assisted SPADs [10]. In this work, the shortcomings from the previous CA-SPAD device were identified and improved. The presented device (CA-SPAD-2) is fabricated in a conventional 350 nm CMOS process.

## 2. Materials and Methods

### Pixel Design and Simulations

**Figure 1:**
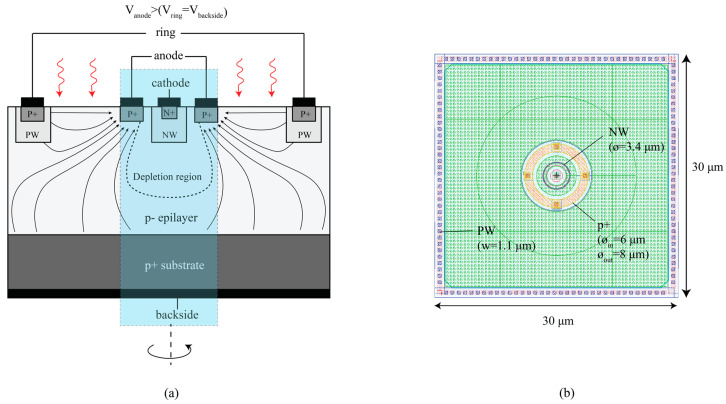
(**a**) Illustration of cross-section of the CA-SPAD-2 device. The depletion region boundary is roughly represented by dotted lines and lines with arrows represent the drift field direction for electrons. The “central SPAD” structure is cylindrical. (**b**) Top-view image of the device from layout with illustrations marking the doping layers and dimensions.

**Figure 2:**
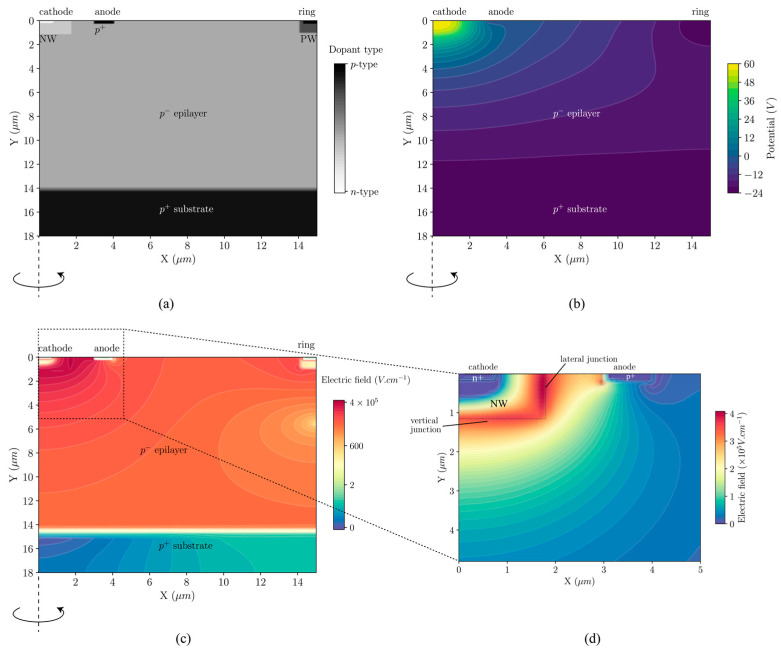
(**a**) Net doping profile of the cross-section of CA-SPAD-2. (**b**) Profile of the potential applied for the simulations. (**c**) Electric field profile (log scale) of the full detector and (**d**) Electric field profile (linear scale) of the central SPAD p-n junction.

**Figure 3:**
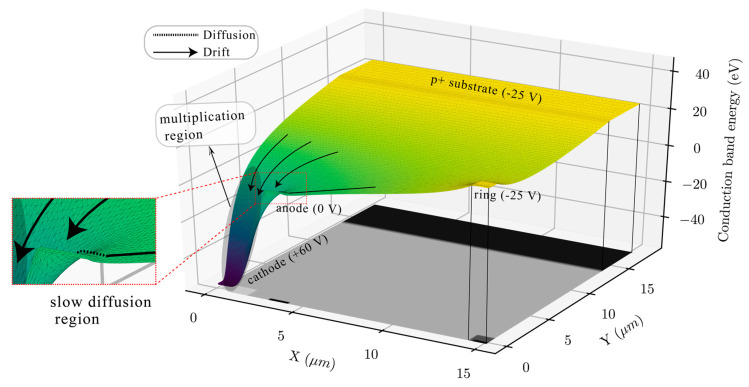
Simulated 3d conduction band energy profile of the CA-SPAD-2 with the 2D net doping profile for reference.

The cross-section of the CA-SPAD-2 device is shown in Figure 1a. The device is fabricated in a cost-effective conventional 350 nm CMOS process from X-fab foundry (XO035 technology). This process has a 14 µm thick epilayer for increased near-infrared (NIR) sensitivity. The epilayer has a high resistivity (~1000 Ω.cm) for reducing power consumption of the detector. Similar to its predecessor, a p+/p-epilayer/Nwell junction is used for the central avalanche region. The Nwell (NW) is preferred over a n+ doping to reduce the number of tunneling electrons which increase the dark count rate. A consequence is the increased breakdown voltage in this type of junction, also aided by the high resistivity lowly-doped epilayer. The Nwell cathode is chosen to be cylindrical and has a lateral width of 3.4 μm. The anode is an annular ring surrounding the cathode and the spacing between the cathode and anode is 1.25 µm. The lateral width of the anode is 1 µm and the central “SPAD” structure has a lateral diameter of 8 µm. The actual area where the avalanche multiplication occurs is much smaller. The distinction between the small multiplication region and the large absorption region can be clearly seen in Figure 2 and Figure 3. The smaller avalanching volume has advantages such as lower excess bias voltages to reach maximum photon detection probability, low junction capacitance, low number of traps and thereby low afterpulsing probability.

In CA-SPAD-1, the square geometry had sharp corners which led to high electric field distribution at the corners and therefore, breakdown only occurred at the sharp corners [15,16] which led to reduced PDP and increased timing jitter [10]. In CA-SPAD-2, the anode and cathode are designed to have a cylindrical geometry to have a uniform electric field distribution around the p-n junction. To create the drift field in the epilayer, a third electrode we call “Ring” is employed. A positive potential difference is applied between the anode and the ring which causes a majority current flow (holes in this case). When a photon is absorbed in the epilayer and generates an electron-hole pair, the electron and the hole are easily separated due to the presence of an electric field. The hole drifts towards the ring and the electron drifts towards the anode. The electron eventually falls into the large depletion volume created by the cathode due to a large reverse voltage. The ring has a square geometry (as shown in Figure 1b) because it provides the maximum fill factor when making arrays, and the ring shape is not critical for the drift field. The p+ substrate is also connected to the ring potential by connecting the backside of the die to the PCB pad through conductive silver paste.

Device physics simulations were performed in Silvaco ATLAS and values for doping were back calculated from the resistivity data from the specifications provided by the foundry. The doping profile of the simulated cylindrical structure can be seen in Figure 2a and the potential applied for the simulation is plotted in Figure 2b. The electric field profile (in log scale) for the whole detector is shown in Figure 2c and a zoomed in electric field profile (in linear scale) at the central SPAD region can be seen in Figure 2d. The lateral junction (indicated in Figure 2d) is the junction of interest, because most photo-electrons arrive from this direction due to the device construction. However, the vertical junction (indicated in Figure 2d) is also capable of initiating an avalanche trigger, since the vertical junction only has slightly lower electric field compared to the lateral junction.

For the same device structure, the conduction band is plotted on a 3D surface plot and can be seen in Figure 3. This 3D plane is analogous to a hill with slopes. A photo-electron generated in the epilayer is analogous to a droplet of water. Given that the waterdrop can never evaporate, it will always move downward. The downward slope leads the drops into the avalanche bucket where they are detected. The waterdrops generated at the surface between the ring and the anode go towards the anode with good speed, slow down near anode because the band is flatter and then slowly diffuse around the anode to fall into the avalanche bucket (illustrated with arrows). The waterdrops in the other regions in the plane have a straight line towards the avalanche bucket and do not experience any delay.

## 3. Results and Discussion

All experiments in this section were performed at room temperature. All measurements are presented in terms of the excess bias voltage (V_ex_), which is the additional bias on top of the breakdown voltage V_bd._ The cathode was biased at V_dd_ (+3 V), the anode at V_dd_-V_bd_-V_ex_ (V_anode_ ~ −46 V) and the ring voltage was maintained at −25 V with respect to anode (V_ring_ = V_anode_−25 V) for high-speed and efficient transport of the photo-electrons. The ring current is ~160 µA for the biasing used. Detailed measurement methods and analysis are described in the Appendix A section.

### 3.1. Breakdown Voltage

**Figure 4:**
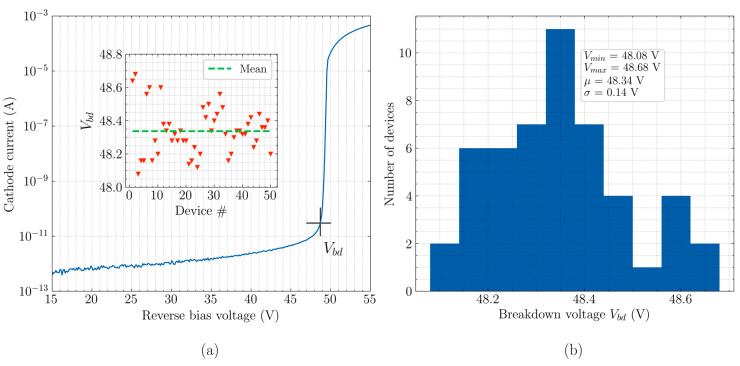
(**a**) IV characteristics of CA-SPAD-2 and (inset)breakdown voltage variation for 50 devices. (**b**) Histogram of breakdown variation for 50 devices.

The IV measurements, to determine the breakdown voltage of the CA-SPAD-2, were performed on a probe station using a HP 4155A semiconductor parameter analyzer and the data is shown in Figure 4a. The breakdown voltage is where the reverse current starts increasing abruptly and in this work, we take the breakdown voltage as the reverse bias voltage at which the reverse current reaches 20 pA (as indicated in Figure 4a). The p-n junction breaks down at around 48.3 V between cathode and anode. The breakdown voltage was measured for the same device located on 50 different dies on the wafer. The variation in breakdown voltage for these devices can be seen in Figure 4a-inset and in the histogram in Figure 4b. The mean of the statistics is 48.34 V with the standard deviation of only 140 mV. Compared to conventional SPADs with large area [17], the breakdown variation is very small. This could be attributed to the relatively small multiplication junction area, since it makes the probability of local non-uniformity/defect due to the fabrication process very low. Conventional SPADs, on the other hand, have larger multiplication junctions and therefore we assume they are more prone to having variations from one device to another on the same wafer.

### 3.2. Passive Quenching Circuit and Re-Triggering

**Figure 5:**
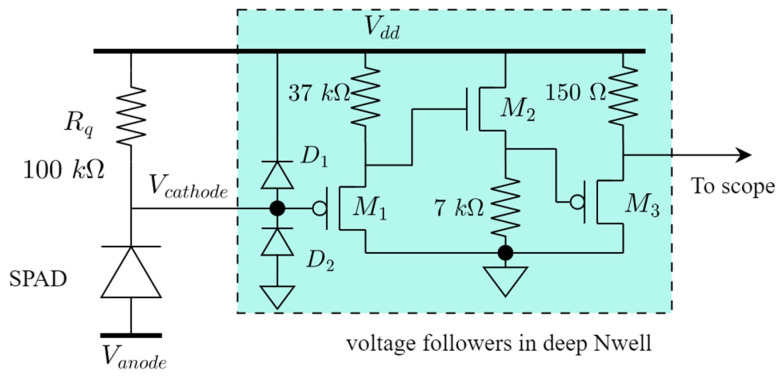
Passive quenching circuit for CA-SPAD-2. The voltage followers are isolated, from the high voltages, by enclosing them in a deep Nwell.

The CA-SPAD-2 device is quenched using a passive quench resistor of 100 kΩ. The CA-SPAD-2 device is located in the p- epilayer. The cathode node is quenched and read-out. Therefore, the cathode is biased at V_dd_ (3 V), the anode is biased at (V_dd_-V_bd_-V_ex_) and the ring is placed at −25 V potential with respect to the anode. The ring is biased at ~ −71 V and therefore, almost all of the p-epilayer is at ~ −71 V. The voltage at cathode is observed through a series of voltage followers (M1, M2 and M3) as illustrated in Figure 5. Transistor M1 has minimum size in order to not introduce too much parasitic capacitance at the cathode. The CA-SPAD-2 diode capacitance is estimated to be around 1fF and the parasitic capacitance due to the traces, the protection diodes D1 and D2 and the gate of M1 is estimated to be ~40 fF. Due to the high voltages in the p- epilayer, the transistors are housed in a Deep Nwell (LDWELL/DNWELL) close to the CA-SPAD-2 (in the same substrate) as illustrated in Figure 5. A bias-tee (Mini-circuits ZFBT-4R2GW+) was used to shift the voltage level to 0 V for compatibility with the Becker and Hickl TCSPC SPC-130 photon counting card used for timing jitter characterization of the CA-SPAD-2.

**Figure 6:**
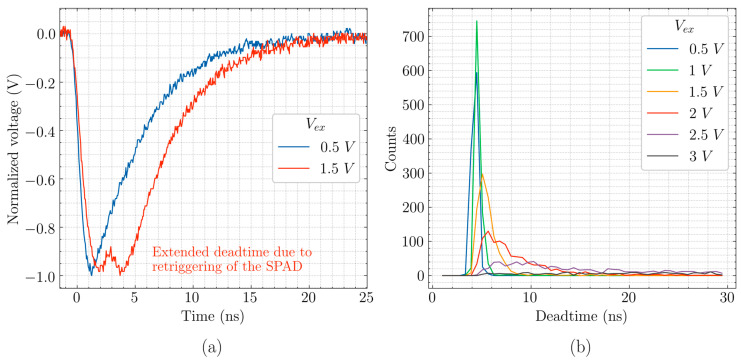
(**a**) Instance of re-triggering behavior observed at excess bias of 1.5 V. (**b**) Histogram of deadtime distribution for various excess bias voltages.

The low capacitance of the CA-SPAD-2 combined with the size of the quenching resistor (100 kΩ) leads to very fast recharging and thereby low deadtimes. But due to this feature, sometimes the CA-SPAD-2 is recharged before all the avalanche carriers are evacuated. These unevacuated carriers trigger another avalanche event and we refer to this phenomenon as “re-triggering”. An instance of re-triggering is shown in Figure 6a. The re-triggering mechanism leads to extended deadtimes (as shown in Figure 6a,b) and the probability that re-triggering occurs is dependent on excess bias voltage (V_ex_). With increasing excess bias voltages, the number of carriers in an avalanche event increases leading to higher probability of re-triggering. The extended deadtimes due to re-triggering has been observed in SPADs with passive quenching circuits [18], and thereby limits the counting rate. This is a main reason why the dark counts saturate close to 3 V excess bias and the PDP drops after 2.6 V excess bias. The pulse with re-triggering can also show up as 2 counts if the detection threshold is set too low. For all the measurements, the detection threshold is set very close to V_dd_ (V_cathode_ in Figure 5 or 0 V in Figure 6a) in order to include the retriggers as a dead-time extension.

### 3.3. Dark Count Rate

**Figure 7:**
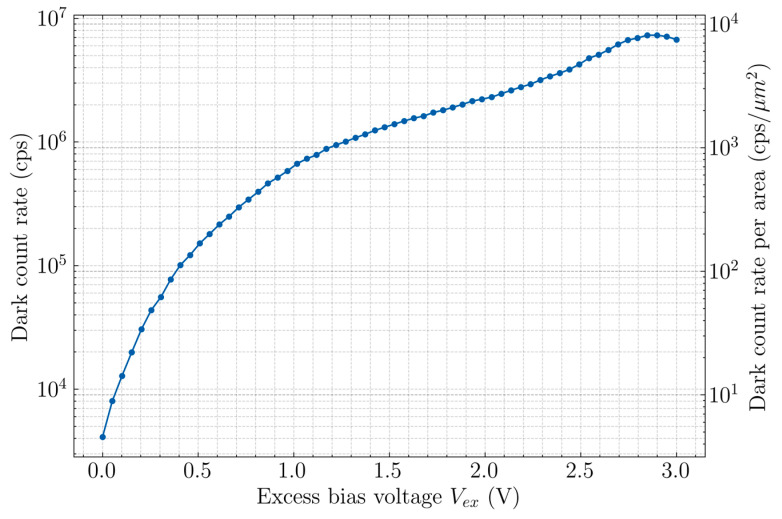
Measured dark count rate for CA-SPAD-2.

A high dark count rate is expected due to the presence of the lateral junction being exposed to the silicon surface. The primary source of the dark counts is tunneling from the p+ anode region. Secondary sources such as surface states and carriers from silicide contact layer also play a part and are more relevant in CA-SPADs due to the lateral junction. The measured dark count rate is shown in Figure 7. The left y-axis is the full dark count rate for a single pixel while the right y-axis is darkcounts per unit area of the device. Close to 3 V V_ex_, the dark count rate starts saturating and then even decreases slightly. This could be attributed to the extended deadtimes at these large excess bias voltages, which can saturate and decrease the counting rates.

### 3.4. Afterpulsing

Afterpulsing is a correlated noise mechanism. There is a given density of traps in a semiconductor, which are atomic defects arising from the fabrication process. These traps can trap free carriers and the trapping probability is proportional to the number of carriers which flow through the trap. A large avalanche current flows through the multiplication junction when there is an avalanche event initiated by a signal carrier. The number of avalanche carriers is proportional the capacitance of the diode, the smaller the diode capacitance, the lesser carriers are required to drop the voltage to V_bd_. If there is a trap in the multiplication region of the SPAD, a carrier can be “trapped” in these traps and be released later in time. The release time, also called trap lifetime, can range from few tens to hundreds of nanoseconds, enough time for the avalanche current to be quenched and the SPAD to be recharged and ready to detect a carrier. Now, when this trapped carrier is released, it will trigger the SPAD and cause a count which is correlated in time with the initial signal photon. These pulses are termed as “afterpulses” and are measured by afterpulsing probability (APP). The trap density in a given semiconductor wafer is fixed. So, the solutions for mitigating afterpulsing in the device level, are limited to reducing the volume of multiplication junction. This has two implications: (1) smaller volume leads to lower traps in that volume and (2) the reduced capacitance also means that the number of carriers passing though the multiplication region during an avalanche pulse is lower and thereby the probability of a carrier getting trapped is reduced. In circuit level, the APP can be kept low by limiting the parasitic capacitance and increasing the deadtime, through active quenching mechanisms, to let the released carrier get collected before the SPAD is active again. But, increasing the deadtime sets a limitation on the maximum attainable count rates.

**Figure 8:**
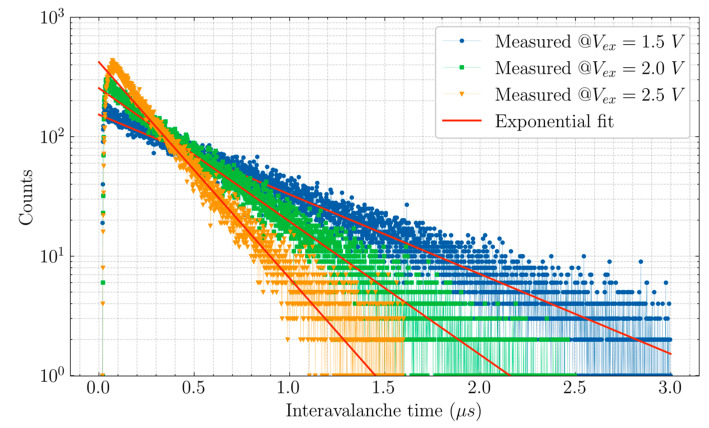
Measured inter-avalanche histogram at excess voltages (V_ex_) = 1.5 V, 2 V and 2.5 V.

One of the methods to determine afterpulsing probability is the inter-avalanche histogram method [19,20,21]. The principle behind that is, from a continuous light source, photons arrive with a Poisson distribution. Also, the dark counts follow the same Poisson distribution. So, if we plot the time of arrival between counts in a histogram, it should ideally follow an exponential distribution. Deviation from this behavior is a result of correlated counts resulting from afterpusling. The deviating counts are referred to as “residuals”. The ratio of the sum of residuals to the sum of total counts gives the afterpulsing probability. The measurement method and analysis is elaborated in the Appendix A. The inter-avalanche histogram for CA-SPAD-2, for different V_ex_, is shown in Figure 8. The APP for CA-SPAD-2 at V_ex_ = 1.5 V, 2 V and 2.5 V are 8%, 10% and 13% respectively. This is possibly a consequence of re-triggering and associated extended deadtimes at V_ex_ = 2.5 V, meaning multiple avalanche triggers take place within a single avalanche pulse and therefore, more charges flow through the multiplication region.

### 3.5. Photon Detection Probability

The sensitivity of SPADs is quantized by the photon detection probability (PDP), usually expressed in%. PDP is used to quantify the sensitivity of single devices and photon detection efficiency (PDE = PDP × Fill factor) is used for quantifying the sensitivity of SPAD devices in arrays. The PDP is the ratio of photon counts of the SPAD to actual number of incident photons. The photon counts of the SPAD is the total amount of counts from the SPAD from which the dark counts are subtracted. The actual number of photons is measured using a reference photodiode. The detailed measurement technique is presented in the Appendix A.

**Figure 9:**
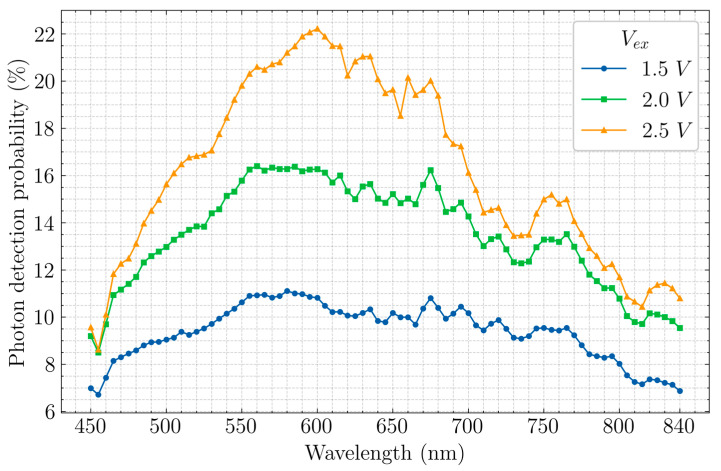
Measured PDP as a function of wavelength for 3 different excess bias voltages.

The measured PDP for 4 different excess biases can be seen in Figure 9. The maximum PDP in this measurement is 22.2% at a wavelength of 600 nm at an excess bias of 2.5 V. It was observed that the maximum PDP is reached at a V_ex_ of ~2.5 V and then the PDP starts decreasing. This is probably caused by the extended deadtimes due to re-triggering (Section 3.2) and saturation happens at high counting rates. The PDP could be further improved by fixing re-triggering which in turn increases counting rate. The PDP at longer wavelengths do not increase with increasing V_ex_, as much as the visible region (~600 nm). This could be due to the vertical junction having lower electric field compared to the lateral junction. The oscillations in the PDP with respect to the wavelength could be attributed to the refractive index difference between the silicon substrate and the silicon dioxide layer above the silicon substrate.

**Figure 10:**
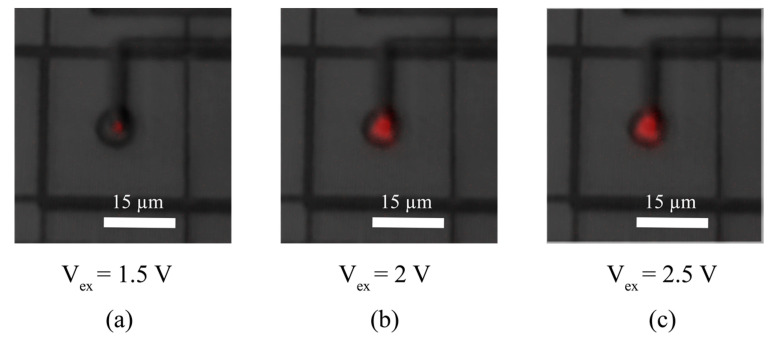
Emission from “central SPAD” area at (**a**) V_ex_ = 1.5 V, (**b**) V_ex_ = 2 V and (**c**) V_ex_ = 2.5 V. The light emission is false-colored red.

The peak PDP at 785 nm for CA-SPAD-1 was 5.6% [10] where the same for CA-SPAD-2 was 12.6%. This improvement is attributed to the cylindrical geometry which improves the symmetry of the avalanching junction. Light emission tests (shown in Figure 10) show one uniform light emission area suggesting that the breakdown occurs uniformly around the junction for CA-SPAD-2 unlike discrete light emission spots of CA-SPAD-1 [10].

### 3.6. Timing Jitter

Timing resolution of SPADs are characterized by timing histograms. The full width half maximum represents the timing jitter of SPADs. Usually conventional SPADs have a timing jitter of a few ps to around 100 ps [22]. This small timing jitter is due to most photons getting absorbed very close to the multiplication region and causing an avalanche instantaneously. However, in CA-SPADs, the photon absorbed farther from the multiplication region and (or) deeper in the epilayer have to transit several (tens of) micrometers before causing an avalanche.

**Figure 11:**
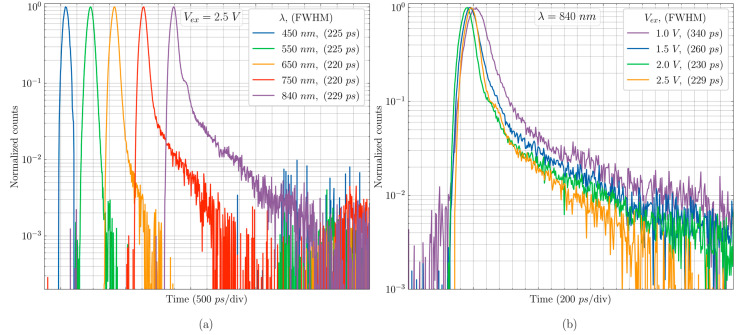
(**a**) Timing histogram measured at an excess bias of 2.5 V for 5 different wavelengths. (**b**) Timing histogram measured at wavelength of 840 nm for 4 different excess bias voltages. The full width half maximum represents timing jitter.

For photon timing measurements, a very sharp (~6 ps) light pulse from a Fianium whitelase micro supercontinuum laser is used and the wavelength is selected through a Fianium superchrome tunable bandpass filter (Bandwidth = 10 nm). The timing histograms are made with a Becker and Hickl SPC-130 TCSPC card where the avalanche pulse (SIGNAL) and the laser sync pulse (SYNC) pulse are compared. CA-SPAD-2 has a timing jitter of 220 ps–230 ps depending on the wavelength and is shown in Figure 11a. CA-SPAD-2′s improved timing resolution compared to CA-SPAD-1 can be attributed to 2 reasons; (1) The detection area is smaller (30 × 30 µm^2^) compared to CA-SPAD-1 (40 × 40 µm^2^) and therefore the farthest distance from the multiplication region is smaller and (2) The electric field is uniform all around the cathode reducing the uncertainty in carrier detection. A slower tail (shown in Figure 11a,b) appears in longer wavelengths due to generation of photo-electrons in the substrate below the epilayer. The timing jitter decreases for increasing V_ex_ as shown in Figure 11b. This is due to lower statistical fluctuation in avalanche build-up [23,24].

The performance parameters of CA-SPAD-2 are summarized in Table 1.

## 4. Conclusions

In summary, we present the improved current-assisted SPAD with on-chip passive quenching circuitry. The device is fabricated in a cost-effective conventional 350 nm CMOS process and therefore the quenching and readout circuitry can be readily made on the same chip in the vicinity of the detector. The transistor circuitry is housed in lowly-doped deep N-well (LDWELL/DNWELL) in order to isolate them from the large operating voltage of the detector. This device shows improvements in all performance parameters compared to its predecessor. Like envisioned, the device shows very good timing response and good photon detection probability in the NIR region. The re-triggering and afterpulsing are challenges which must be addressed in order to improve the PDP. With back-side illumination (BSI) technology, the CA-SPADs can be scaled to much smaller pixel pitches (<10 µm) and a much deeper epilayer (>20 µm), where a vertical drift field is applied, for improved NIR response.

## Figures and Tables

**Table 1:** Performance parameters of this CA-SPAD-2.

Technology	350 nm
Detection area (shape)	30 × 30 µm^2^ (square)
p-n junction type	p+/p- epi/N-well
p-n junction geometry	cylindrical
Breakdown voltage	48.34 V ^1^
Excess bias voltage (V_ex_)	2.5 V ^1^
Timing jitter, FWHM [λ]	220 ps, [650 nm]
PDP_max_, [λ]	22.2%, [600 nm]
Afterpulsing probability	~13%
Quenching type	On-chip passive quenching (100 kΩ)
Ring current	160 µA ^1^
Power/pixel	4 mW ^1^

^1^ −25 V ring voltage with respect to anode voltage.

**Figure A1:**
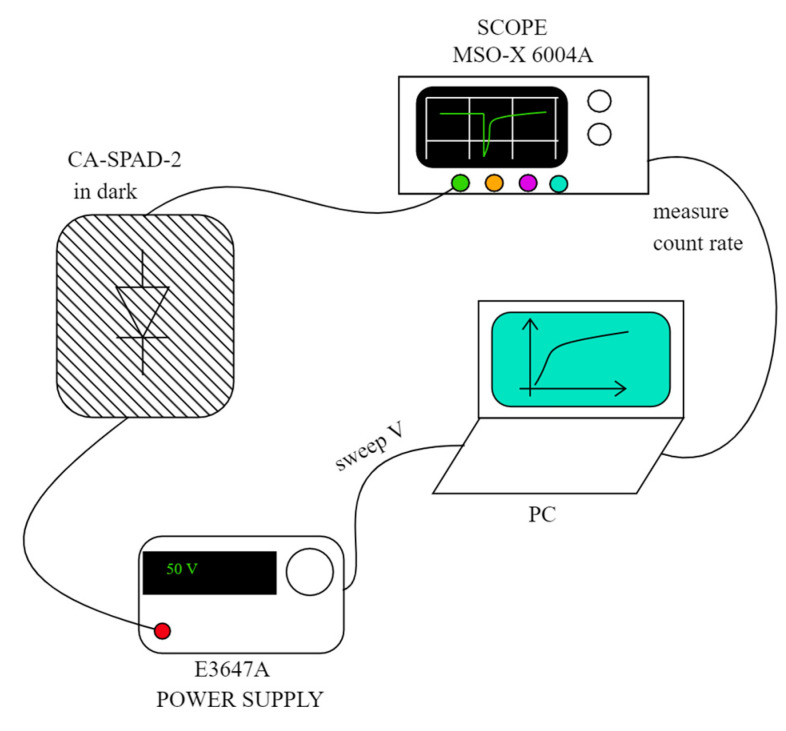
DCR measurement setup.

**Figure A2:**
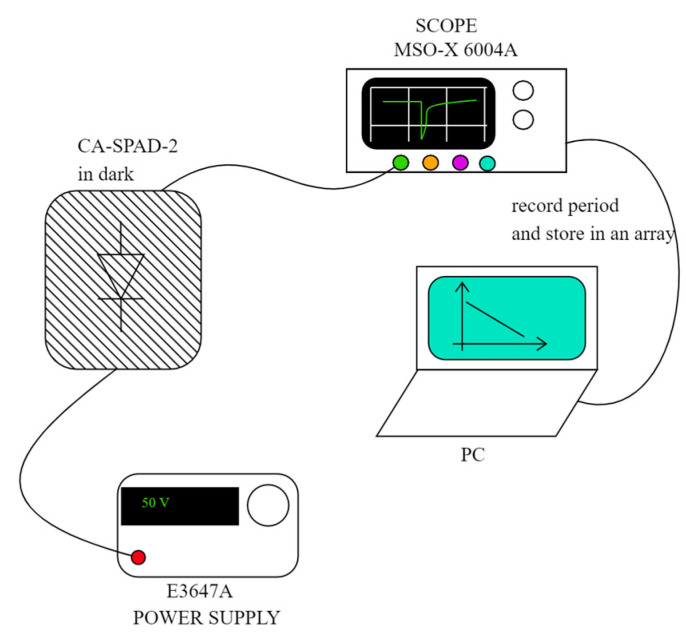
Interavalanche time measurement setup.

**Figure A3:**
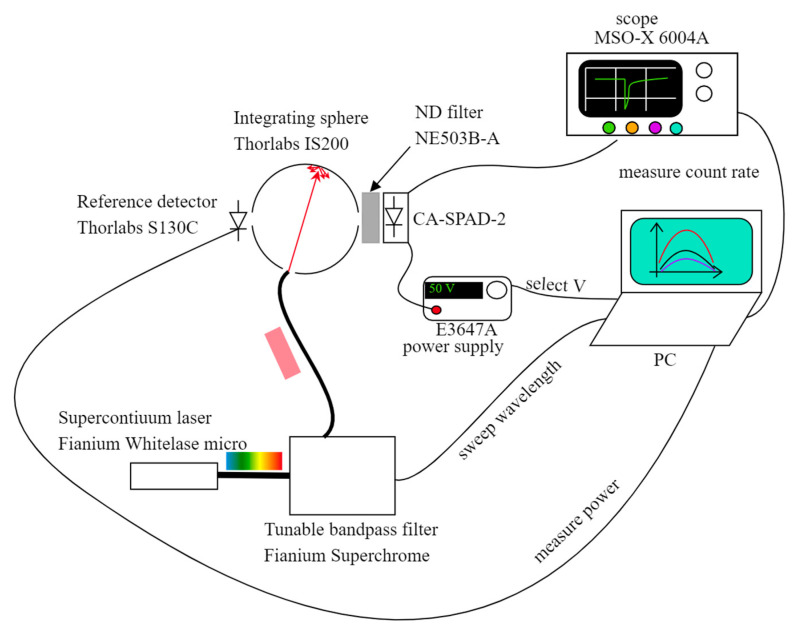
PDP measurement setup.

## Appendix A

### Appendix A.1. Dark Count Rate

The oscilloscope was triggered on the avalanche pulse itself where the trigger level was set very close to the top of the pulse (for instance near 0 V in Figure 6a) to avoid re-triggers. For the dark count rate measurements, the device under test (DUT) was kept dark while the counts are sampled using the counter function in the Oscilloscope. The measurement setup is shown in Figure A1.

### Appendix A.2. Afterpulsing

For the afterpulsing measurement, a scope is triggered on the avalanche pulses. The time from the triggered pulse to the consecutive pulse is measured and added in a histogram. The measured period is the interavalanche time and the measurement setup for measuring the interavalanche times is shown in Figure A2.

100,000 data points were taken for Figure 8. The parameters for the histogram are given in Table A1.

**Table A1:** Histogram details.

V_ex_	Range	Bin Width
1.5 V	3 µs	1 ns
2.0 V	2.5 µs	1 ns
2.5 V	1.5 µs	1 ns

The fit was made with Equation (A1) where *t* is the interavalanche time. To find the values for *a* and *b,* the tail part of the interavalanche time curve is fed to the SciPy’s optimize function [25]. The tail part is chosen because afterpulsing contributions tend to be lower with larger interavalanche times. The fit parameters calculated by SciPy optimize are given in Table A2. The constant *b* corresponds with the dark count rate measured in Figure 7 [26].

(A1) $$fit=a*e^{-b*t}$$

The difference between the exponential fit and the measured data is calculated, and we refer to the differences as “residuals”. The afterpulsing probability (APP) is the ratio of the sum of residuals to the sum of the total counts in the measured data.

**Table A2:** Fit parameters calculated by Scipy optimize and the calculated APP.

V_ex_	a	b	APP (in%)
1.5 V	153	1.54 × 10^6^	8
2.0 V	255	2.57 × 10^6^	10
2.5 V	421	4.17 × 10^6^	13

### Appendix A.3. Photon Detection Probability

The setup for PDP measurement is shown in Figure A3. The CA-SPAD-2 was biased at a certain V_ex._ The detector is kept dark and dark counts (per second) (#dark counts) were measured using an oscilloscope. For illuminating the detector uniformly, an integrating sphere with 3 ports was used. A supercontinuum laser (Fianium whitelase micro) with a tunable bandpass filter (Fianium Superchrome) was used as the light source. The DUT and a reference power meter were placed at the two exit ports. There was a neutral density filter in between the exit port of the integrating sphere and the CA-SPAD-2 to prevent saturation (the attenuation factor is included in the calculations). The wavelength was swept, and the total counts from the CA-SPAD-2 (# total counts) and the optical power from the reference detector were recorded. The number of photons (# of photons) were calculated from the optical power. The PDP was calculated through Equation (A2). APP is afterpulsing probability.

(A2) $$\mathrm{PDP}=\frac{\left(\#\;\mathrm{total}\;\mathrm{counts}-\#\mathrm{dark}\;\mathrm{counts}\right)*\left(1-\mathrm{APP}\right)}{\#\;\mathrm{of}\;\mathrm{photons}}$$

### Appendix A.4. Light Emission Test

A CA-SPAD-2 device without any quench resistor was biased above breakdown voltage so that, when the avalanche initiates, a large current keeps flowing through the junction. This leads to very inefficient light emission and this light emission is recorded using a Thorlabs CS165MU1—Zelux™ 1.6 MP Monochrome CMOS Camera with a 50× objective. Since the intensity of light emission is relatively low, the gain of the camera sensor was chosen to be highest (gain = 48) and the exposure time was 5 s. This emission image is overlaid with a second image of the illuminated CA-SPAD-2. To provide contrast, the light emission is false-colored red.

### Appendix A.5. Data Analysis

Python was used to analyze and plot data. NumPy [27], Pandas [28] and Matplotlib [29] were used for number analysis and plotting. “SciencePlots” plotting style was used (https://github.com/garrettj403/SciencePlots) [30].

Python library Scipy’s [25] optimize function was used for fitting data for Afterpulsing analysis. Python library Pyvisa (https://pyvisa.readthedocs.io/en/latest/) was used to communicate with the oscilloscope and the voltage source. Python library Pywinauto (https://pywinauto.readthedocs.io/en/latest/) was used to include wavelength sweep function in the PDP measurements.
